# Supplementary material for: Assessment of Rapid MinION Nanopore DNA Virus Meta-Genomics Using Calves Experimentally Infected with Bovine Herpes Virus-1
Source: Viruses. 2022 Aug 24;14(9):1859. doi: 10.3390/v14091859 (PMC9501177; doi:10.3390/v14091859)
Supplement: Supplementary file 1 [file viruses-14-01859-s001.zip › Figure S1.pdf]

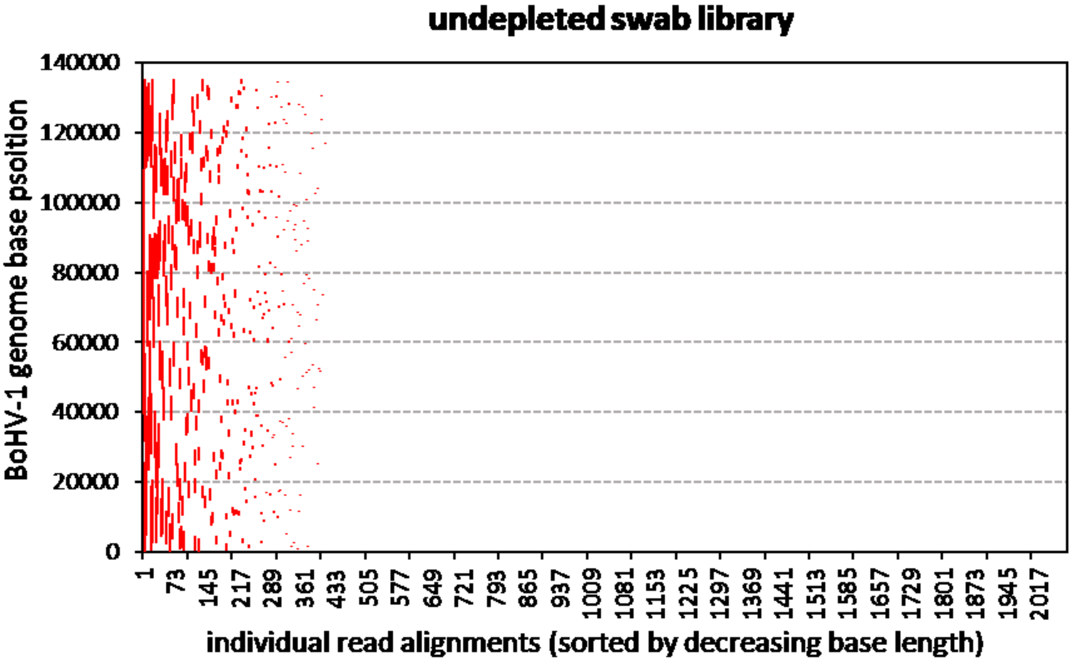

Average alignment length = 4,013. Number of aligned bases = 1,657,348. Number of aligned reads = 414.
Total bases sequenced = 1,800 Mb. % of total bases sequenced that aligned to BoHV-1 genome = 0.092%

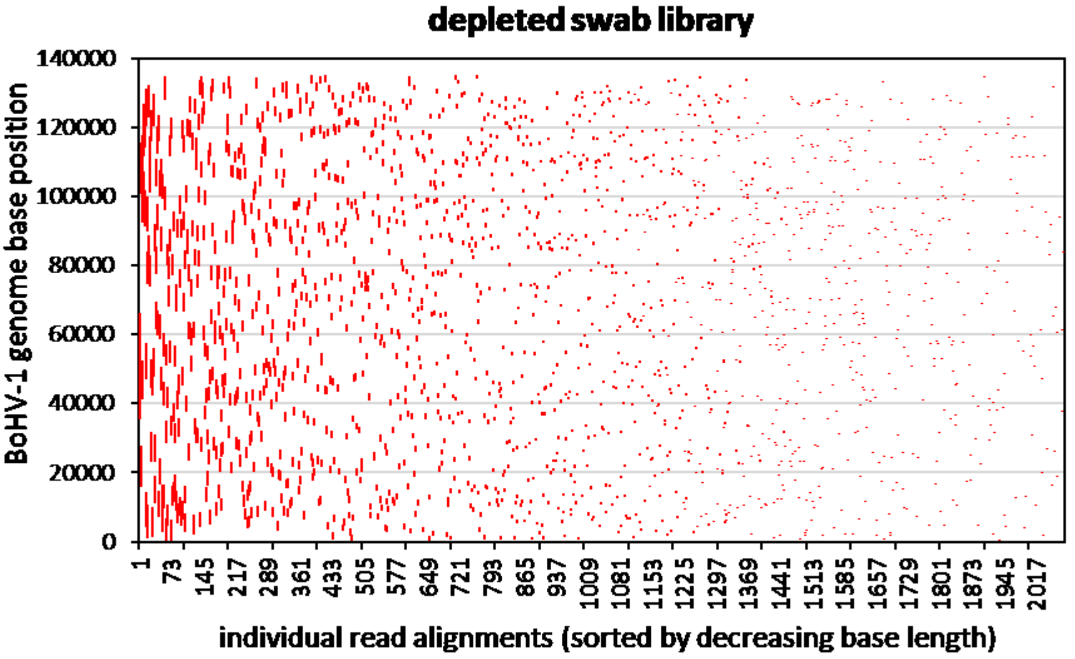

Average alignment length = 1,443. Number of aligned bases = 3,003,635. Number of aligned reads = 2,082.
Total bases sequenced 85.7 Mb. % of total bases sequenced that aligned to BoHV-1 genome = 3.5%

Figure S1. Alignments to the BoHV-1 genome of sequence from undepleted and depleted (bead-beating and nuclease treatment prior to nucleic acid extraction) libraries generated from a nasal swab from a calf infected with BoHV-1. PCR-free tagmented libraries were generated with the ONT Field Sequencing Kit and sequenced on a MinION R9 flowcell using rapid base calling. FASTQ files were aligned to the BoHV-1 genome sequence using the EPI2ME Custom Reference Aligner workflow which employs minimap2. Graphs were created in Microsoft Excel and Microsoft PowerPoint in Microsoft Office Professional Plus 2016.
