# Supplementary material for: Assessment of Rapid MinION Nanopore DNA Virus Meta-Genomics Using Calves Experimentally Infected with Bovine Herpes Virus-1
Source: Viruses. 2022 Aug 24;14(9):1859. doi: 10.3390/v14091859 (PMC9501177; doi:10.3390/v14091859)
Supplement: Supplementary file 1 [file viruses-14-01859-s001.zip › Figure S3.pdf]

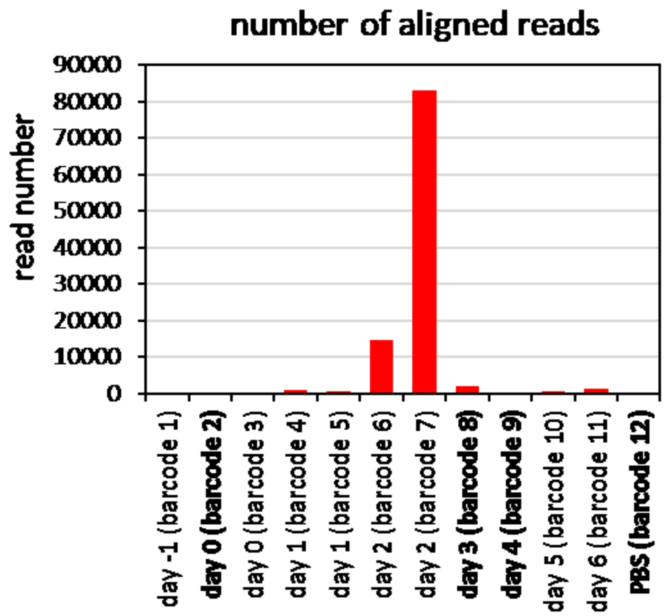

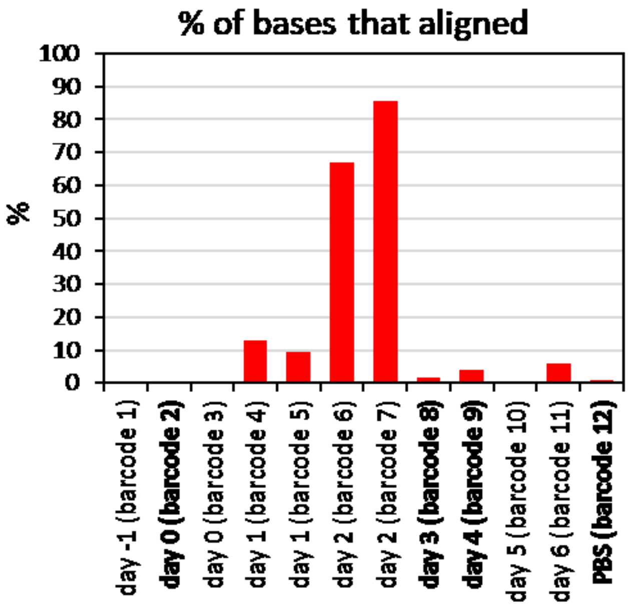

Figure S3. Comparison of consistency between sequence library duplicates when either read counts or the percent of bases aligning to the BoHV-1 genome are used. This shows wide variation between BoHV-1 read number between samples with different barcodes but consistency between duplicate libraries of swabs taken on day 1 (barcode 4 and 5) and day 2 (barcodes 6 and 7) of the challenge of day when the number of bases that aligned to the BoHV-1 genome were calculated as a percentage of all bases sequenced. Graphs were created in Microsoft Excel and Microsoft PowerPoint in Microsoft Office Professional Plus 2016.
