# Supplementary material for: Assessment of Rapid MinION Nanopore DNA Virus Meta-Genomics Using Calves Experimentally Infected with Bovine Herpes Virus-1
Source: Viruses. 2022 Aug 24;14(9):1859. doi: 10.3390/v14091859 (PMC9501177; doi:10.3390/v14091859)
Supplement: Supplementary file 1 [file viruses-14-01859-s001.zip › Table S1.pdf]

	Source	Origin	Characteristics	Culture Medium	Reference
**Bovine foetal lung cells**	AFBI, Veterinary Science Division, Stormont, Belfast, N. Ireland	Bovine foetal lungs	Semi-continuous cells, 23 passages, negative for *Mycoplasma* spp. and BVDV	G-MEM 2%	Negative
**Bovine foetal lung cells infected with BoHV-1-1-1-1**	AFBI, Veterinary Science Division, Stormont, Belfast, N. Ireland	BoHV-1-1-1-1 strain 2011-415 wt isolated from the trachea of a calf with IBR diagnosis	5 mL of a dilution 1:100 of BoHV-1-1-1-1 in G-MEM 2% (MOI=1) were used to infect foetal lung cells	G-MEM 2%	Positive

Table S1. Details of bovine foetal cell lung cultures used for infection with BoHV-1.
